# Coordination of histone chaperones for parental histone segregation and epigenetic inheritance

**DOI:** 10.1101/gad.351278.123

**Published:** 2024-02-01

**Authors:** Yimeng Fang, Xu Hua, Chun-Min Shan, Takenori Toda, Feng Qiao, Zhiguo Zhang, Songtao Jia

**Affiliations:** 1 Department of Biological Sciences, Columbia University, New York, New York 10027, USA;; 2 Institute for Cancer Genetics, Columbia University, New York, New York 10027, USA;; 3 Department of Pediatrics, Columbia University, New York, New York 10027, USA;; 4 Department of Genetics and Development, Columbia University Irving Medical Center, New York, New York 10032, USA;; 5 State Key Laboratory of Plant Genomics, Institute of Microbiology, Chinese Academy of Sciences, Beijing 100101, China;; 6 Department of Biological Chemistry, School of Medicine, University of California, Irvine, Irvine, California 92697, USA

**Keywords:** heterochromatin, H3K9 methylation, epigenetic inheritance, histone chaperone, Mcm2, Dpb3, Dpb4, eSPAN, fission yeast, parental histone density

## Abstract

Chromatin-based epigenetic memory relies on the accurate distribution of parental histone H3–H4 tetramers to newly replicated DNA strands. Mcm2, a subunit of the replicative helicase, and Dpb3/4, subunits of DNA polymerase ε, govern parental histone H3–H4 deposition to the lagging and leading strands, respectively. However, their contribution to epigenetic inheritance remains controversial. Here, using fission yeast heterochromatin inheritance systems that eliminate interference from initiation pathways, we show that a Mcm2 histone binding mutation severely disrupts heterochromatin inheritance, while mutations in Dpb3/4 cause only moderate defects. Surprisingly, simultaneous mutations of Mcm2 and Dpb3/4 stabilize heterochromatin inheritance. eSPAN (enrichment and sequencing of protein-associated nascent DNA) analyses confirmed the conservation of Mcm2 and Dpb3/4 functions in parental histone H3–H4 segregation, with their combined absence showing a more symmetric distribution of parental histone H3–H4 than either single mutation alone. Furthermore, the FACT histone chaperone regulates parental histone transfer to both strands and collaborates with Mcm2 and Dpb3/4 to maintain parental histone H3–H4 density and faithful heterochromatin inheritance. These results underscore the importance of both symmetric distribution of parental histones and their density at daughter strands for epigenetic inheritance and unveil distinctive properties of parental histone chaperones during DNA replication.

## Abstract (precis)

In this study, Fang et al. report that the helicase subunit MCM2, DNA polymerase ε subunits DPB3/4, and histone chaperone FACT complex function collectively to maintain symmetrical parental histone segregation during heterochromatin replication in yeast. They further reveal a basis for leading/lagging strand bias of heterochromatin inheritance and describe the distinct dependence of inheritance efficiency on histone density, providing new insight into the mechanisms of inheritable epigenetic memory.

Eukaryotic genomic DNA is folded with histones to form nucleosomes, which serve as the fundamental units of chromatin. Covalent modifications of histones play essential roles in establishing gene expression programs that determine cell identity (Escobar et al. 2021; Du et al. 2022). Histones with certain modifications, especially those associated with repressed states, can be passed down to subsequent generations even after the initial signals for the recruitment of histone-modifying enzymes have disappeared, thereby creating an epigenetic memory (Escobar et al. 2021; Du et al. 2022). The prevailing view is that during DNA replication, the passage of the replication fork disrupts parental nucleosomes, and parental H3–H4 tetramers with pre-existing modifications are equally deposited on both daughter strands at their original locations to guide nucleosome formation (Xu et al. 2010; Gan et al. 2018; Petryk et al. 2018; Yu et al. 2018; Escobar et al. 2019; Schlissel and Rine 2019). In addition, newly synthesized H3–H4, which lack parental histone modifications, also direct nucleosome formation to fill in the gaps created by DNA duplication. The existing modifications on parental histone H3–H4 recruit corresponding modifying enzymes, leading to modifications of nearby newly synthesized histone H3–H4, thereby restoring the original histone modification patterns on both replicated chromatids (Fig. 1A; Escobar et al. 2021; Du et al. 2022).

**Figure 1.**
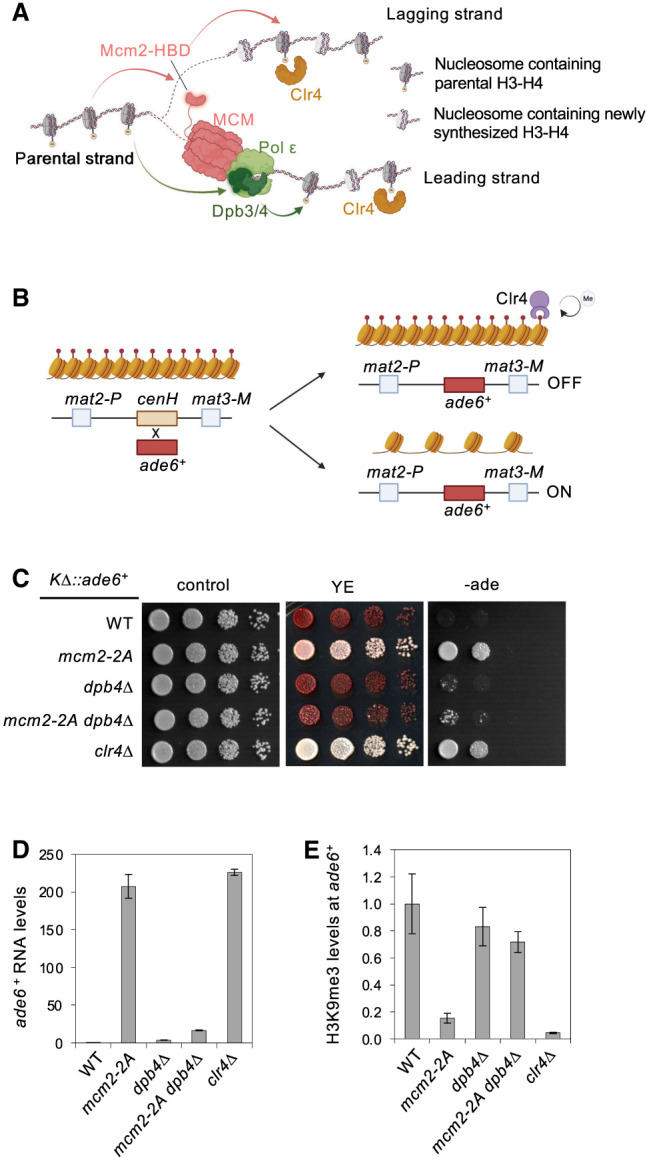
Mcm2 and Dpb3/4 regulate the inheritance of mating-type region heterochromatin. (*A*) Schematic diagram of parental histone H3–H4 segregation pathways during DNA replication. (*B*) Schematic diagram of the *KΔ::ade6*^+^ reporter. (*C*) Serial dilution analysis of the indicated strains to measure the expression of *KΔ::ade6*^+^. (*D*) qRT-PCR analyses of *ade6*^+^ transcript levels, normalized to *act1*^+^. Data are presented as mean ± SD of three technical replicates. (*E*) ChIP-qPCR analysis of H3K9me3 level at *KΔ::ade6*^+^, normalized to *act1*. Data are presented as mean ± SD of three technical replicates.

Recent advancements in eSPAN (enrichment and sequencing of protein-associated nascent DNA) have provided important insights into the mechanisms controlling parental histone distribution during DNA replication (Yu et al. 2014, 2018; Gan et al. 2018; Petryk et al. 2018; Li et al. 2020). The findings collectively reveal that Mcm2, a component of the replicative helicase, is responsible for transferring parental histone H3–H4 to the lagging strand. Conversely, Dpb3/Dpb4 (POLE4/POLE3 in mammals), part of the leading strand polymerase ε, are responsible for transferring parental histone H3–H4 to the leading strand (Fig 1A).

One critical remaining question is how the misregulation of parental histone segregation affects epigenetic inheritance. According to the aforementioned model, mutations of either Mcm2 or Dpb3/4 histone binding activities are expected to severely impact epigenetic inheritance. However, these mutations have surprisingly mild effects on budding yeast and fission yeast heterochromatin, which has been studied extensively as a heritable chromatin state (He et al. 2017; Yu et al. 2018; Saxton and Rine 2019). In mammalian systems, although defective parental histone segregation affects stem cell maintenance and differentiation, only subsets of the epigenome are affected (Li et al. 2020; Xu et al. 2022; Tian et al. 2023; Wen et al. 2023; Wenger et al. 2023).

One possible reason for the subtle effects of Mcm2 or Dpb3/4 mutations on the epigenome is the existence of additional parental histone segregation pathways. The histone chaperone FACT (facilitates chromatin transcription) is involved in nucleosome disassembly and reassembly during gene transcription, DNA replication, and DNA repair (Formosa and Winston 2020). FACT is required for heterochromatin integrity, which has been attributed to its role in regulating histone turnover and interacting with the heterochromatin machinery (Lejeune et al. 2007; Holla et al. 2020; Murawska et al. 2021; Takahata et al. 2021). Recently, it has been shown that budding yeast FACT has a role in parental histone transfer (Wang et al. 2023). However, how FACT is engaged in parental histone segregation during DNA replication remains largely unknown.

Another possible reason is the existence of sequence-dependent pathways that initiate chromatin states regardless of epigenetic inheritance. Therefore, it is critical to examine epigenetic inheritance without the influence of initiation pathways. In the fission yeast *Schizosaccharomyces pombe*, heterochromatin primarily forms at repetitive DNA elements in the pericentric region, subtelomeres, and silent mating-type region (Grewal and Jia 2007). Nucleosomes within these regions are dimethylated and trimethylated at histone H3 lysine 9 (H3K9me2 and H3K9me3, respectively), which recruits HP1 family proteins to repress transcription (Thon and Verhein-Hansen 2000; Bannister et al. 2001; Nakayama et al. 2001b; Hall et al. 2002; Sadaie et al. 2004). Importantly, initiation and inheritance pathways can be effectively separated (Allshire and Madhani 2018; Grewal 2023; Shan et al. 2023). During the initiation step, the histone H3K9 methyltransferase Clr4 is recruited to specific genomic loci through the RNA interference (RNAi) machinery or DNA binding proteins to initiate H3K9me3. During the subsequent inheritance step, Clr4 restores heterochromatin structure after DNA replication by recognizing pre-existing histone modifications independent of the recruitment signal. The chromodomain of Clr4 plays a crucial role in this process by recognizing H3K9me3, facilitating Clr4 recruitment to parental histones (Zhang et al. 2008). Clr4 then modifies nucleosomes formed by newly synthesized histones during and after DNA replication to restore H3K9me3 levels, known as the “read–write” coupling.

The ability to genetically separate the two steps makes fission yeast heterochromatin assembly a superb model for studying the mechanism of epigenetic inheritance (Shan et al. 2023). For example, at the silent mating-type region (MTR), *cenH* recruits Clr4 through the RNAi pathway (Hall et al. 2002; Jia et al. 2004). When *cenH* is replaced by a reporter gene, de novo initiation is mostly disabled, resulting in the reporter exhibiting one of two states: expressed or silenced (Grewal and Klar 1996). The silenced state is efficiently maintained through both mitosis and meiosis, clearly demonstrating epigenetic inheritance. In addition, an ectopic heterochromatin inheritance system is developed by recruiting Clr4 to *tetO* binding sites through a TetR-Clr4-SET domain (TetR-Clr4-I) fusion protein (Audergon et al. 2015; Ragunathan et al. 2015). The addition of tetracycline removes TetR-Clr4-I from *tetO*, abolishing any additional initiation events. In the absence of histone H3K9 demethylase Epe1, this ectopic heterochromatin is inherited by subsequent generations through endogenous Clr4. These assays allow precise measurements of heterochromatin inheritance without interference from initiation.

In this study, we used these inheritance-specific assays to demonstrate the crucial role of Mcm2-mediated parental histone H3–H4 deposition to the lagging strand for epigenetic inheritance, whereas Dpb3/4-mediated deposition of parental histone H3–H4 to the leading strand makes a comparatively minor contribution. Importantly, simultaneous mutations of Mcm2 and Dpb3/4 result in stable epigenetic inheritance and reduced parental histone segregation bias, suggesting the importance of a symmetric distribution of parental histones in epigenetic inheritance as well as the involvement of additional histone chaperones in regulating this process. Specifically, we discovered that FACT regulates parental histone H3–H4 segregation independently of DNA strands and collaborates with Mcm2 and Dpb3/4 to maintain parental histone H3–H4 levels. Moreover, we demonstrated that both the symmetric distribution of parental histone H3–H4 and their density at individual daughter strands collectively determine the outcomes of heterochromatin inheritance. Our findings establish a critical link between parental histone segregation and epigenetic inheritance and highlight the cooperative roles of distinct histone chaperone activities in regulating epigenetic inheritance.

## Results

### Mcm2 and Dpb3/4 have distinct roles in regulating epigenetic inheritance

To examine the role of parental histone segregation in epigenetic inheritance in fission yeast, we first tested whether the functions of the histone chaperones involved in parental histone segregation in budding yeast and mammals—Mcm2 and Dpb3/4—are conserved in fission yeast (Fig. 1A). We generated a GST-tagged version of the histone binding domain of Mcm2 (Mcm2-HBD, amino acids 1–110) and a mutant version containing mutations of two conserved tyrosine residues to alanine (Y81A and Y89A; referred to here as Mcm2-2A) (Supplemental Fig. S1A; Foltman et al. 2013; Huang et al. 2015), which abolish binding of budding yeast and mammalian Mcm2-HBD to the H3–H4 tetramer. We also generated recombinant fission yeast H3 and H4 and assembled H3–H4 tetramers. GST pull-down analyses show that GST-Mcm2-HBD, but not GST or GST-Mcm2-HBD-2A, interacts with H3–H4 tetramers (Supplemental Fig. S1B). Although Dpb3 and Dpb4 are difficult to produce alone as recombinant proteins, we were able to generate a GST-Dpb4/6xHis-Dpb3 heterodimer through the coexpression of GST-Dpb4 and 6xHis-Dpb3, followed by two-step purifications with Talon and GST resins. The purified GST-Dpb3/Dpb4 complex also interacted with H3–H4 tetramers in a GST pull-down assay (Supplemental Fig. S1B). These results suggest that fission yeast Mcm2 and Dpb3/4 interact with H3–H4 tetramers, similar to other systems.

To examine the role of the histone binding activity of Mcm2 in vivo, we introduced the *mcm2-2A* mutation and a C-terminal 3xFLAG tag at the endogenous *mcm2*^+^ locus. Western blot analysis shows that Mcm2-2A is expressed at levels comparable with those of wild-type Mcm2 (Supplemental Fig. S1C). We also generated *dpb3Δ* and *dpb4Δ*, which remove the open reading frames of *dpb3*^+^ and *dpb4*^+^, respectively. We then assessed the effects of *mcm2-2A* and *dpb4Δ* on the inheritance-specific reporter *KΔ::ade6*^+^, which was created by replacing the *cenH* sequence at the silent mating-type region (MTR) with an *ade6*^+^ gene (Fig. 1B). Loss of *cenH* disrupts heterochromatin initiation at the MTR, and *KΔ::ade6*^+^ can only be maintained as one of two epigenetic states: “*ade6-on*” or “*ade6-off*.” Heterochromatin at the MTR is lost in “*ade6-on*” cells, resulting in white colonies on a low-adenine medium (YE) and robust growth on a medium without adenine (−ade). In contrast, heterochromatin at the MTR is properly inherited in “*ade6-off*” cells, leading to red colonies on YE medium and poor growth on −ade medium. We used *KΔ::ade6*^+^ cells with the “*ade6-off*” state as a starting point to examine the epigenetic inheritance of heterochromatin. As expected, *clr4Δ* cells form white colonies on YE medium and grow robustly on −ade medium (Fig. 1C). Strikingly, *mcm2-2A* and *dpb4Δ* show distinct phenotypes. *mcm2-2A* cells produce light-pink/white colonies on YE medium and show robust growth on −ade medium, indicating a severe loss of heterochromatin inheritance (Fig. 1C; Supplemental Fig. S1D). In contrast, *dpb4Δ* cells form red colonies on YE medium and exhibited minimal growth on −ade medium, indicating efficient heterochromatin inheritance (Fig. 1C). Notably, the absence of *KΔ::ade6*^+^ inheritance defects observed in *dpb4Δ* cells is not due to functional redundancy between Dpb3 and Dpb4, as both *dpb3Δ* and *dpb3Δ dpb4Δ* cells show similar phenotypes (Supplemental Fig. S2A). Interestingly, *mcm2-2A dpb4Δ* and *mcm2-2A dpb3Δ* cells form red colonies on YE medium and exhibit poor growth on −ade medium (Fig. 1C; Supplemental Fig. S2A), suggesting that *dpb3Δ* or *dpb4Δ* suppresses the effects of *mcm2-2A* on heterochromatin inheritance. Consistent with dilution analysis results, qRT-PCR analyses reveal much higher *ade6*^+^ transcript levels in *mcm2-2A* cells, similar to *clr4Δ* cells. In contrast, *dpb4Δ* and *mcm2-2A dpb4Δ* cells display only slightly higher *ade6*^+^ transcript levels than wild-type cells (Fig. 1D). Furthermore, chromatin immunoprecipitation (ChIP) analyses demonstrate that H3K9me3 levels at *KΔ::ade6*^+^ are nearly abolished in *mcm2-2A* and *clr4Δ* cells, are only mildly affected in *dpb4Δ* cells, and are at intermediate levels in *mcm2-2A dpb4Δ* cells (Fig. 1E). Collectively, these findings highlight the involvement of Mcm2 and Dpb3/4 in heterochromatin inheritance.

**Figure 2.**
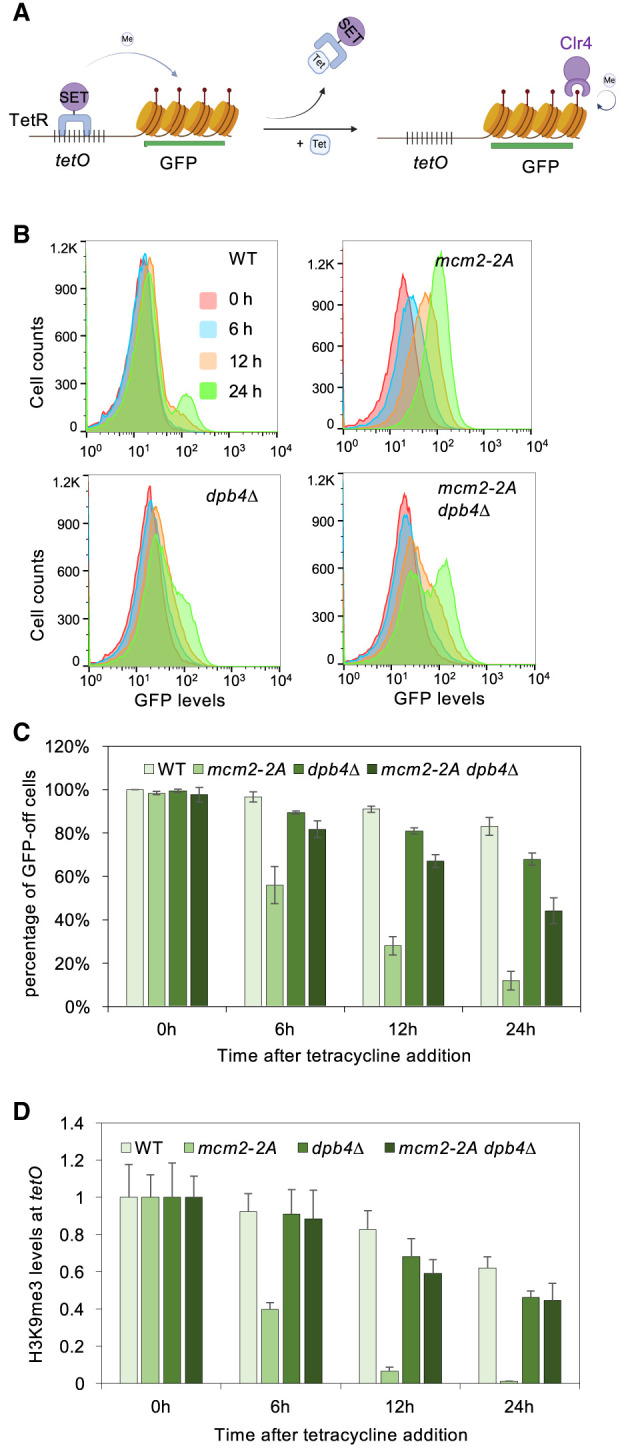
Mcm2 and Dpb4 regulate the inheritance of an ectopic heterochromatin. (*A*) Schematic diagram of the *tetO-gfp*^+^ reporter. (*B*) Flow cytometry analysis of GFP expression at different time points after tetracycline addition. All strains used are in an *epe1Δ* background. (*C*) Quantification of the percentage of cells maintaining low GFP expression at different time points after tetracycline addition. Data are presented as mean ± SD of two biological replicates. All strains are in an *epe1Δ* background. (*D*) ChIP-qPCR analysis of H3K9me3 levels at *tetO* binding sites at different time points after tetracycline addition, normalized to *act1*. Data are presented as mean ± SD of three technical replicates. All strains are in an *epe1Δ* background.

To further investigate the impact of Mcm2 and Dpb3/4 on epigenetic inheritance, we examined the effects of their mutations on the inheritance of an ectopic heterochromatin. In this system, 10 copies of the *tetO* binding sites along with a *gfp*^+^ reporter gene are inserted into the endogenous *ura4*^+^ locus (Fig. 2A; Ragunathan et al. 2015). A Clr4 SET domain is fused with the TetR protein to create TetR-Clr4-I, which is recruited to the *tetO* binding sites, leading to the formation of an H3K9me3 domain and subsequent silencing of the *gfp*^+^ reporter. TetR-Clr4-I can be quickly disassociated from the *tetO* binding sites by the addition of tetracycline to prevent further initiation. The existing heterochromatin is expected to self-sustain through the action of endogenous Clr4, which binds H3K9me3 through its chromodomain and propagates it through the “read and write” cycle when the H3K9 demethylase Epe1 is absent. Therefore, all strains used for this analysis are in an *epe1Δ* background.

To examine the inheritance of this ectopic heterochromatin with high temporal resolution, we used flow cytometry to monitor GFP expression levels (Fig. 2B,C; Supplemental Fig. S2B). The majority of wild-type cells maintain a “GFP-off” state 24 h (∼10 cell generations) following tetracycline addition, with only a minor fraction of cells showing a “GFP-on” state. However, *mcm2-2A* cells exhibit fast activation of GFP expression compared with *dpb4Δ* cells, which show only very slow activation of GFP expression. Additionally, *mcm2-2A dpb4Δ* cells show a mixture of “GFP-off” and “GFP-on” states. Furthermore, ChIP analysis confirms that wild-type cells maintain H3K9me3 levels at the *tetO* binding sites 24 h after tetracycline addition. However, H3K9me3 levels decline quickly in *mcm2-2A* cells. Conversely, H3K9me3 exhibits slower decay in both *dpb4Δ* and *mcm2-2A dpb4Δ* cells (Fig. 2D). The similarities observed in the phenotypic patterns between the *KΔ::ade6*^+^ and *tetO-gfp*^+^ systems support the notion that Mcm2 exerts a stronger influence on epigenetic inheritance compared with Dpb3/4. Furthermore, the simultaneous disruption of both Mcm2 and Dpb3/4 leads to reasonably efficient heterochromatin inheritance, suggesting that these two histone-binding proteins function in an antagonistic manner.

### Mcm2 and Dpb3/4 have minor roles in pericentric heterochromatin inheritance

**Figure 3.**
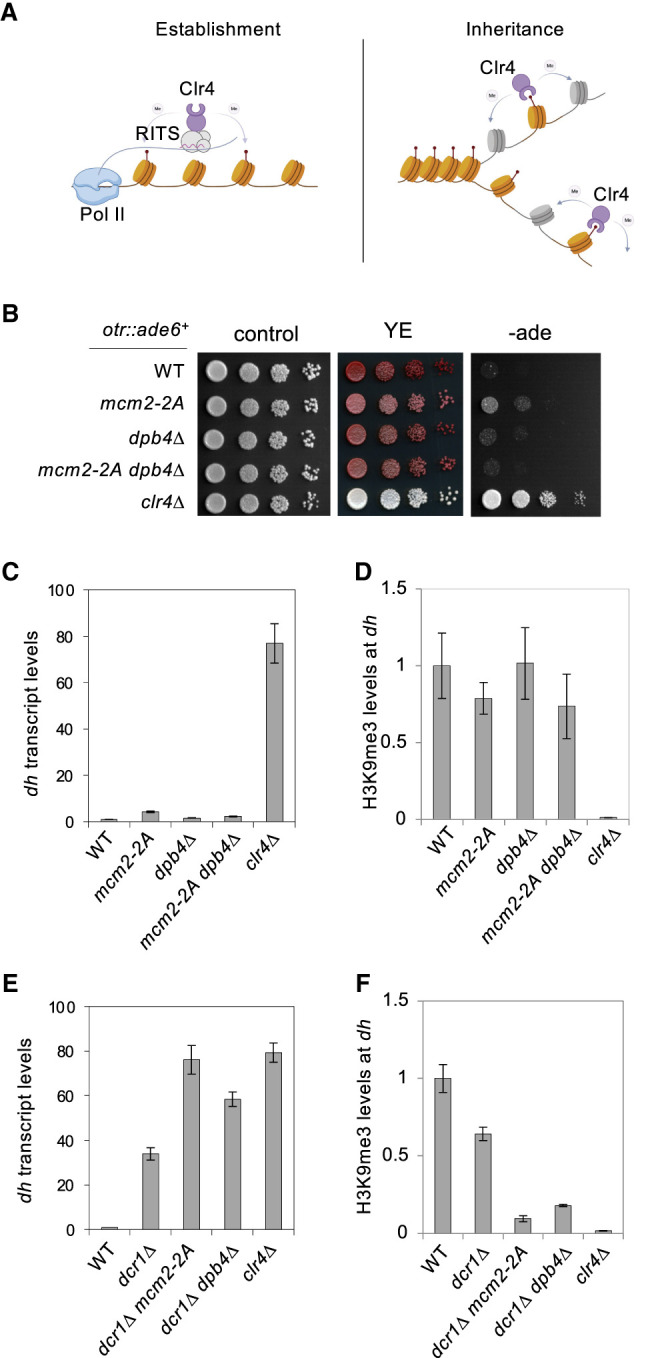
Mcm2 and Dpb4 cooperate with RNAi to regulate pericentric heterochromatin. (*A*) Schematic diagram of heterochromatin establishment and inheritance pathways at the pericentric region. (*B*) Serial dilution analysis of the indicated strains to measure the expression of *otr::ade6*^+^. (*C*,*E*) qRT-PCR analysis of *dh* transcript levels, normalized to *act1*^+^. Data are presented as mean ± SD of three technical replicates. (*D*,*F*) ChIP-qPCR analysis of H3K9me3 level at *dh*, normalized to *leu1*. Data are presented as mean ± SD of three technical replicates.

We also investigated the roles of Mcm2 and Dpb3/4 in regulating pericentric heterochromatin integrity, where the *dh* and *dg* repeats initiate heterochromatin formation through the RNAi pathway (Fig. 3A; Volpe et al. 2002; Verdel et al. 2004). We used the *otr::ade6*^+^ reporter, where an *ade6*^+^ gene is inserted within the pericentric repeats. In *clr4Δ* cells, loss of heterochromatin leads to the formation of white colonies on YE medium and strong growth on −ade medium (Fig. 3B). In contrast, *mcm2-2A* cells give rise to dark-pink colonies on YE medium and show mild growth on −ade medium, while *dpb4Δ*, *dpb3Δ*, and *mcm2-2A dpb4Δ* cells produce red colonies on YE medium and show poor growth on −ade medium (Fig. 3B; Supplemental Fig. S2C). Consistent with the observed phenotypes, qRT-PCR analyses show a slight increase of *dh* transcript levels in *mcm2-2A* cells, while *dpb4Δ* and *mcm2-2A dpb4Δ* cells exhibit marginally higher *dh* transcript levels than wild-type cells (Fig. 3C). Moreover, ChIP analyses show that *mcm2-2A*, *dpb4Δ*, and *mcm2-2A dpb4Δ* cells exhibit very minor reduction of H3K9me3 levels compared with wild-type cells (Fig. 3D). It is important to note that their impact on *dh* transcript levels and H3K9me3 levels at *dh* are very minor compared with the drastic effects of *clr4Δ*.

The above results suggest a minor role of Mcm2 and Dpb3/4 on pericentric heterochromatin integrity. One possible reason is that RNAi-mediated heterochromatin initiation preserves pericentric heterochromatin integrity even when heterochromatin inheritance is compromised. To test this idea, we examined the requirement of Mcm2 and Dpb3/4 on pericentric heterochromatin integrity when RNAi is compromised. The ribonuclease Dicer (Dcr1) is a critical component of the RNAi pathway and is required for heterochromatin establishment at pericentric repeats (Volpe et al. 2002; Verdel et al. 2004). qRT-PCR analyses show that *dh* transcripts levels increase in *dcr1Δ* cells and further increase in *dcr1Δ mcm2-2A* and *dcr1Δ dpb4Δ* cells (Fig. 3E). Moreover, ChIP analyses show that H3K9me3 levels at *dh* repeats decrease in *dcr1Δ* cells but further decrease in *mcm2-2A dcr1Δ* and *dpb4Δ dcr1Δ* cells (Fig. 3F). These findings suggest that RNAi and Mcm2-Dpb3/4 cooperate to regulate the integrity of pericentric heterochromatin.

The *dpb4Δ* mutant displays more pronounced effects on pericentric heterochromatin in the *dcr1Δ* background compared with the *KΔ::ade6*^+^ and *tetO-gfp*^+^ systems. Despite the expectation that *dcr1Δ* primarily affects heterochromatin establishment, it causes robust silencing defects at pericentric repeats as a result of a higher histone turnover rate, mediated by factors such as Epe1, the Mst2 histone acetyltransferase, the INO80 chromatin remodeling complex, and the Paf1 complex (Reddy et al. 2011; Aygün et al. 2013; Sadeghi et al. 2015; Wang et al. 2015b; Shan et al. 2020). Therefore, *dcr1Δ* might create a more sensitized background for the effects of *dpb4Δ* to be observed.

We also directly tested the effects of Mcm2 and Dpb3/4 on heterochromatin establishment by using genetic crosses to introduce the *otr::ade6*^+^ reporter from a *clr4Δ* background (Supplemental Fig. S3). We found that neither *mcm2-2A* nor *dpb4Δ* exhibits any difference in the silencing status of *otr::ade6*^+^ compared with strains in which *otr::ade6*^+^ is introduced from a *clr4*^+^ background. These results demonstrate that Mcm2 and Dpb3/4 do not affect heterochromatin establishment.

### Mcm2 and Dpb3/4 regulate parental histone H3–H4 segregation during DNA replication

**Figure 4.**
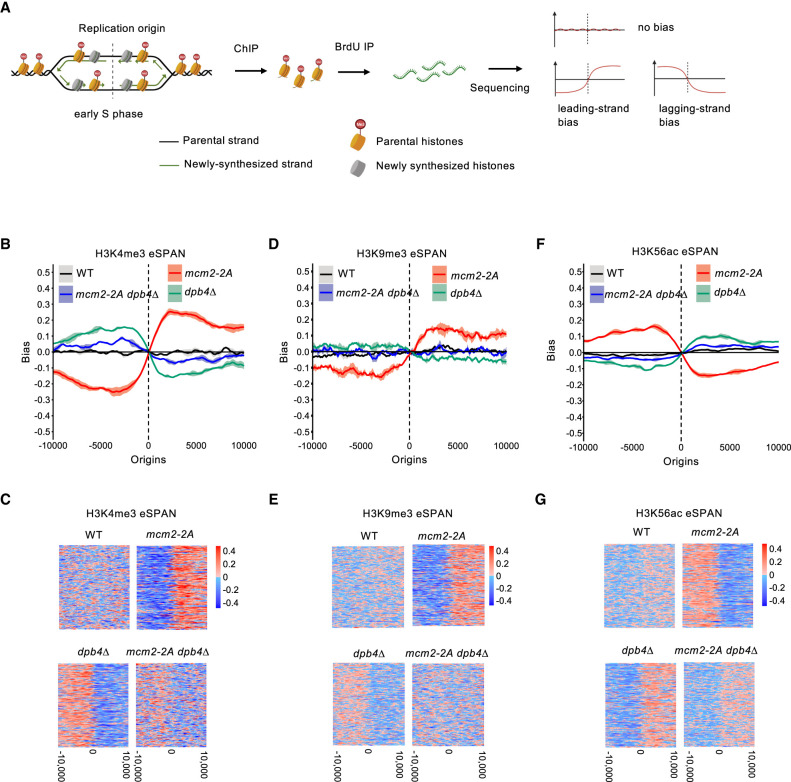
Mcm2 and Dpb4 regulate parental histone H3–H4 deposition at the replication fork. (*A*) Schematic diagram of eSPAN workflow and expected results. (*B*,*D*,*F*) eSPAN analysis of H3K4me3, H3K9me3, or H3K56ac bias levels at replication origins. The shading of the bias line plot is the 95% confidence interval of mean value of at least two biological replicates, which is mean ± twofold of the standard error. (*C*,*E*,*G*) Heat maps of H3K4me3, H3K9me3, and H3K56 eSPAN bias at each of the 162 replication origins analyzed.

We then investigated the molecular mechanisms through which Mcm2 and Dpb3/4 influence heterochromatin inheritance. Considering their roles as regulators of parental histone segregation in budding yeast and mammals, we examined their impact on parental histone segregation during DNA replication in fission yeast using eSPAN (Fig. 4A; Yu et al. 2014, 2018; Gan et al. 2018). We first synchronized yeast cultures at the G2/M phase by growing a *cdc25-22* temperature-sensitive mutant at restrict temperature. After release into permissive temperature, BrdU was added before S phase to label newly synthesized DNA (Hodson et al. 2003; Sivakumar et al. 2004), and cells were fixed at early S phase (Supplemental Fig. S4A). All mutants used had a similar septation index profile (Supplemental Fig. S4A) and were not sensitive to hydroxyurea (HU) (Supplemental Fig. S4B), a ribonucleotide reductase inhibitor, indicating that they did not have major DNA replication defects. Chromatin immunoprecipitation (ChIP) was then performed using antibodies against H3K4me3 or H3K9me3, representing parental histones in euchromatin and heterochromatin, respectively. A subsequent immunoprecipitation with an antibody against BrdU allowed us to the isolate newly replicated single-strand DNA (ssDNA) for sequencing. We calculated the partition ratio of Watson (*W*) and Crick (*C*) strands with the formula

$$\left(\frac{W-C}{W+C}\right)$$

at selected replication origins to determine the eSPAN bias (Fig. 4A; Yu et al. 2014, 2018; Gan et al. 2018). To ensure methodological consistency, we selected 162 replication origins that exhibited consistent usage in the wild-type samples and had been annotated as origins in the Pombase for our analysis.

We initially focused on examining the segregation of H3K4me3-containing parental histones during DNA replication due to its high enrichment at replication origins. While wild-type cells displayed no H3K4me3 eSPAN bias, *mcm2-2A* cells exhibited a strong leading strand bias, and *dpb4Δ* cells showed a strong lagging strand bias (Fig. 4B,C). These results align with previous findings in budding yeast and mammals (Gan et al. 2018; Petryk et al. 2018; Yu et al. 2018; Li et al. 2020), highlighting the conservation of Mcm2 and Dpb3/4 in regulating parental histone deposition. Interestingly, *mcm2-2A dpb4Δ* cells showed reduced bias levels compared with each of the single mutants (Fig. 4B,C).

Next, we examined the segregation of H3K9me3-containing parental histones during DNA replication. As H3K9me3 is not typically enriched at replication origins, the data were less robust. Nevertheless, H3K9me3 eSPAN in different genetic backgrounds displayed a pattern similar to that of H3K4me3 eSPAN, indicating that the segregation of parental histone H3–H4 was likely independent of the modifications they carried (Fig. 4D,E). Considering the more robust H3K4me3 eSPAN results, we focused on H3K4me3 eSPAN for our subsequent analyses of parental histone H3–H4.

To validate our findings, we also investigated the segregation of newly synthesized histones, which are enriched with acetylated histone H3 lysine 56 (H3K56ac) (Masumoto et al. 2005). The H3K56ac eSPAN results show that wild-type cells did not exhibit bias, *mcm2-2A* cells displayed a lagging strand bias, and *dpb4Δ* cells showed a leading strand bias. Additionally, *mcm2-2A dpb4Δ* cells showed a reduction in bias, approaching levels similar to wild-type cells (Fig. 4F,G). The H3K56ac eSPAN results were the opposite of those of H3K4me3 eSPAN, suggesting that defects in parental histone transfer are partially compensated for by the deposition of newly synthesized histones.

Collectively, these results demonstrate that Mcm2 and Dpb3/4 are critical for parental histone H3–H4 segregation to the lagging and leading strands, respectively, highlighting the conservation of parental histone segregation pathways.

### Parental histone H3–H4 density is critical for epigenetic inheritance.

**Figure 5.**
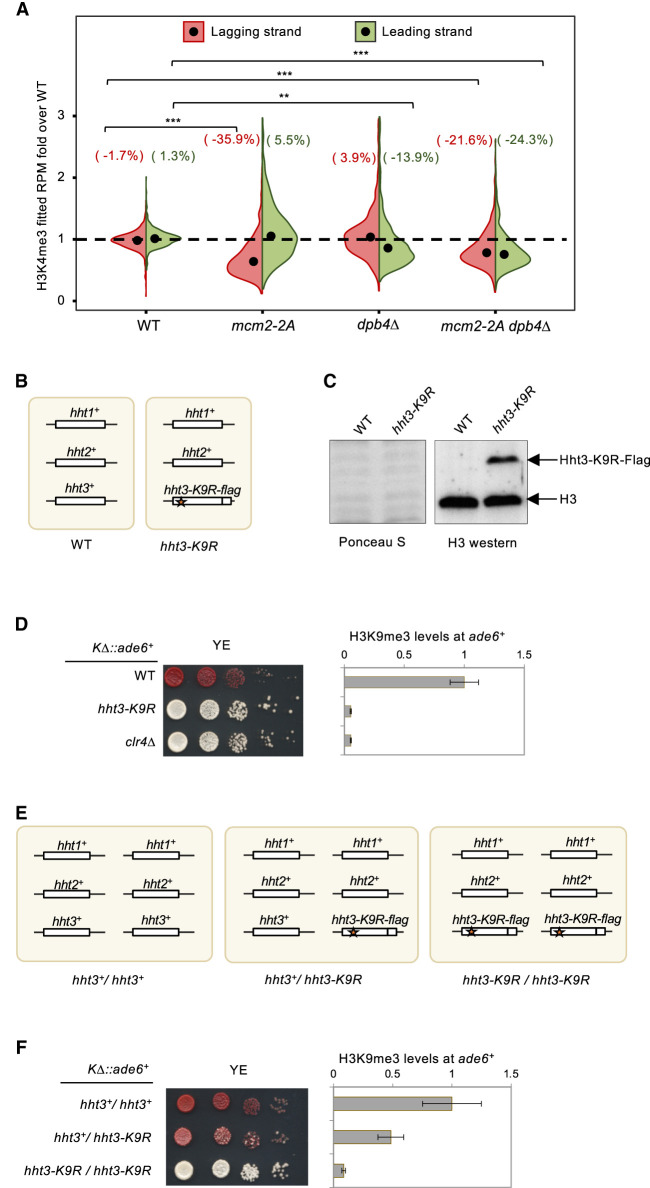
Maintaining parental histone H3–H4 density on daughter strands during DNA replication is critical for epigenetic inheritance. (*A*) Violin plot of H3K4me3 density on the leading and lagging strands at replication origins. Data are the average of at least two biological repeats for each genotype. The numbers represent change over WT for each strand. (*B*,*E*) Diagram of histone H3 genes and the *hht3-K9R* mutation. (*C*, *left*) Ponceau S stain of the membrane used for Western blot analysis. (*Right*) Western blot analysis of histone H3 levels in cell extracts. Hht3-K9R has a FLAG tag at its C terminus, resulting in a higher molecular weight. (*D*,*F*, *left*) Serial dilution analysis of the indicated strains to measure the expression of *KΔ::ade6*^+^. (*Right*) ChIP analysis of H3K9me3 levels at *KΔ::ade6*^+^, normalized to *leu1*. Data are presented as mean ± SD of three technical replicates.

To further investigate the connections between parental histone H3–H4 segregation and epigenetic inheritance of heterochromatin, we analyzed parental histone density on daughter strands for each genotype. To make a quantitative comparison, we first normalized raw H3K4me3 eSPAN coverage to the total number of reads of each sample and then normalized against BrdU input to derive a density score. The normalized density value for each genotype was then divided by the corresponding wild-type (WT) density to determine the fold change. In wild-type cells, the daughter strands exhibited similar H3K4me3 density, consistent with balanced parental histone H3–H4 deposition (Fig. 5A). In *mcm2-2A* cells, the lagging strand displayed a strong decrease in H3K4me3 density, while the leading strand showed a slight increase. Conversely, in *dpb4Δ* cells, the leading strand showed a decrease in H3K4me3 density, while the lagging strand remained almost unchanged. Interestingly, the *mcm2-2A dpb4Δ* double mutant demonstrated a reduction in H3K4me3 density on both strands, consistent with the disruption of both deposition pathways (Fig. 5A).

We then investigated whether directly modulating the density of parental histones containing H3K9me3 affects heterochromatin inheritance. In fission yeast, H3 is encoded by three genes: *hht1*^+^, *hht2*^+^, and *hht3*^+^. We introduced a K9R mutation together with a FLAG tag into *hht3*^+^ (*hh3-K9R*), which is expected to result in about one-third of histones lacking H3K9me3 (Fig. 5B). Western blot analysis with an H3 antibody shows that H3K9R is ∼36% ± 6% (*n* = 3) of total histone H3 (Fig. 5C). As expected, this mutation impaired the silencing of *KΔ::ade6*^+^ and the inheritance of *tetO-gfp*^+^ after TetR-Clr4-I release (Fig. 5D; Supplemental Fig. S5), consistent with the idea that high parental histone density is required for proper epigenetic inheritance. Importantly, *hht3-K9R* only marginally affects pericentric heterochromatin (Shan et al. 2016), suggesting that it does not significantly affect heterochromatin initiation.

To further determine the histone density threshold required for proper heterochromatin inheritance, we generated diploids with either one or two copies of *hht3-K9R* (Fig. 5E). When two copies of *hht3-K9R* were present, there were severe silencing defects of *KΔ::ade6*^+^, as indicated by white colonies on YE medium and a strong reduction of H3K9me3 levels at the reporter. In contrast, the presence of one copy of *hht3-K9R* exhibited only mild silencing defects of *KΔ::ade6*^+^, marked by mostly red colonies on YE medium and an intermediate decrease in H3K9me3 levels (Fig. 5F). Therefore, we conclude that the parental histone threshold required for heterochromatin inheritance falls within the range of 64%–82%. This threshold also aligns with the observed relationship between silencing defects and H3K4me3 density in our eSPAN analysis.

### FACT regulates parental histone H3–H4 segregation to both daughter strands

The observation that *mcm2-2A dpb4Δ* cells exhibit considerable leading strand H3K4me3 eSPAN bias suggests the potential contribution of other histone chaperones to parental histone deposition when both Mcm2 and Dpb3/4 are absent. We turned our focus to the histone chaperone complex FACT, which is involved in nucleosome dynamics during DNA replication (Formosa and Winston 2020).

**Figure 6.**
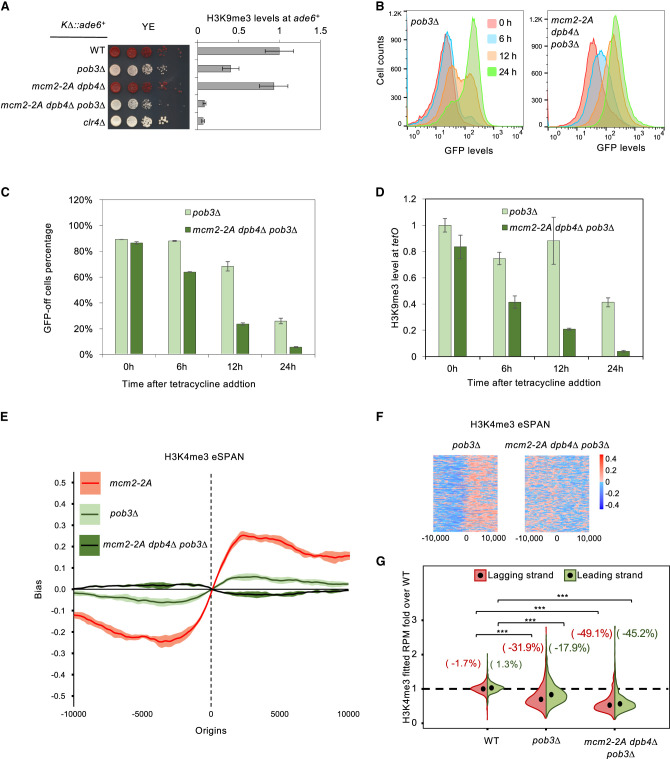
FACT regulates parental histone deposition and heterochromatin inheritance. (*A*, *left*) Serial dilution analysis of the indicated genotypes to measure the expression of *KΔ::ade6*^+^. (*Right*) ChIP-qPCR analysis of H3K9me3 levels *KΔ::ade6*^+^, normalized to *leu1*. Data are presented as mean ± SD of three technical replicates. (*B*) Flow cytometry analysis of GFP expression at different time points after tetracycline addition. All strains are in an *epe1Δ* background. (*C*) Quantification of the percentage of cells maintaining low GFP expression at different time points after tetracycline addition. Data are presented as mean ± SD of two biological replicates. All strains are in an *epe1Δ* background. (*D*) ChIP-qPCR analysis of H3K9me3 levels at *tetO* at different time points after tetracycline addition, normalized to *leu1*. Data are presented as mean ± SD of three technical replicates. All strains are in an *epe1Δ* background. (*E*) eSPAN analysis of H3K4me3 bias levels at replication origins of the indicated strains. The shading of the bias line plot is the 95% confidence interval of mean value of at least two biological replicates, which is approximately mean ± twofold of the standard error. (*F*) Heat maps of H3K4me3 bias at each of the 162 replication origins analyzed. (*G*) Violin plot of H3K4me3 density on leading and lagging strands at replication origins. Data are the average of at least two biological repeats for each genotype. The numbers represent changes over WT for each strand.

The yeast FACT complex consists of Spt16 and Pob3 (Formosa and Winston 2020). While Spt16 is essential for cell survival, *pob3Δ* is viable for further genetic analysis. The inheritance of *KΔ::ade6*^+^ is compromised in *pob3Δ* cells, as evidenced by light-pink colonies on YE medium, and is further compromised in *mcm2-2A dpb4Δ pob3Δ* cells, resulting in white colonies on YE medium (Fig. 6A). ChIP analyses confirmed that H3K9me3 levels at *KΔ::ade6*^+^ are reduced in *pob3Δ* cells and further decrease in *mcm2-2A dpb4Δ pob3Δ* cells (Fig. 6A). Similarly, *pob3Δ* cells showed a quick activation of GFP expression and loss of H3K9me3 at *tetO* after tetracycline addition to remove TetR-Clr4-I. In addition, *mcm2-2A dpb4Δ pob3Δ* cells showed an even faster rate of GFP activation and H3K9me3 loss at *tetO* compared with *pob3Δ* cells (Fig. 6B–D).

We then investigated the role of FACT in parental histone segregation. H3K4me3 eSPAN revealed a mild leading strand bias in *pob3Δ* cells, and *mcm2-2A dpb4Δ pob3Δ* cells displayed even milder H3K4me3 eSPAN bias, although directed toward the lagging stand (Fig. 6E,F). However, *pob3Δ* cells showed strong reductions in histone density on both strands, and *pob3Δ mcm2-2A dpb4Δ* cells displayed an even more pronounced reduction in density on both strands (Fig. 6G). Similar to what has been observed in other mutant backgrounds, H3K56 eSPAN mirrors that of H3K4me3 eSPAN (Supplemental Fig. S6). These findings suggest that the FACT complex acts in concert with Mcm2 and Dpb3/4 for parental histone segregation and heterochromatin inheritance.

## Discussion

One of the central questions of chromatin-based epigenetic inheritance is how parental histone H3–H4 tetramers are distributed to daughter strands, which serve as seeds to restore parental histone modification patterns. The histone binding activities of two replisome components, Mcm2 and Dpb3/4, are responsible for distributing parental histone H3–H4 to the lagging and leading strands, respectively. However, despite their strong effects on parental histone H3–H4 segregation, mutations of Mcm2 and Dpb3/4 have only mild effects on heterochromatin stability in yeast (Foltman et al. 2013; He et al. 2017; Yu et al. 2018; Saxton and Rine 2019). In this study, we investigated the role of effects of mutating the histone binding activity of Mcm2 (*mcm2-2A*) and Dpb3/4 (*dpb3Δ* or *dpb4Δ*) in heterochromatin inheritance in fission yeast using inheritance-specific reporter assays. Surprisingly, *mcm2-2A* abolishes heterochromatin inheritance, *dpb3Δ* and *dpb4Δ* have only minor defects, and *mcm2-2A dpb3Δ* and *mcm2-2A dpb4Δ* have intermediate defects. We also found that these mutants have mild effects on native pericentric heterochromatin because initiation and inheritance pathways function together to regulate pericentric heterochromatin integrity, and Mcm2 and Dpb3/4 are only required for heterochromatin inheritance. These results align with recent studies in mammalian systems indicating that the disruption of parental histone H3–H4 segregation pathways can impact cellular processes in specific contexts, potentially due to defective epigenetic inheritance (Li et al. 2020, 2023; Xu et al. 2022; Tian et al. 2023; Wen et al. 2023; Wenger et al. 2023).

We also developed eSPAN in fission yeast to measure parental histone H3–H4 segregation. We found that H3K4me3 eSPAN and H3K9me3 eSPAN generally agree with each other, suggesting that the segregation of parental histone H3–H4 is independent of their modifications. This is consistent with the fact that histone chaperones, such as Mcm2-HBD, interact with the core histone fold rather than histone tails, where these modifications occur (Huang et al. 2015; Richet et al. 2015; Wang et al. 2015a). We observed that H3K9me3 eSPAN is less robust compared with H3K4me3 eSPAN, which we attribute to much higher levels of H3K4me3 than H3K9me3 around replication origins. However, given the interaction between heterochromatin proteins and parental histone chaperones within the DNA replication machinery (Nakayama et al. 2001a; Motamedi et al. 2008; Fischer et al. 2009; Takahata et al. 2021; Li et al. 2023), it is conceivable that differences may arise in the deposition of H3K9me3-containing parental histones, particularly in specialized contexts. For example, in mouse embryonic stem cells, H3K9me3 is preferentially transferred to the leading strand at long interspersed nuclear element (LINE) retrotransposons, mediated by the interaction of the human silencing hub (HUSH) complex and DNA polymerase ε (Li et al. 2023).

We also performed H3K56ac eSPAN to measure the deposition of newly synthesized histone H3–H4. Interestingly, it anticorrelated with H3K4me3 eSPAN. These results confirm that our procedure effectively distinguishes histone segregation into the two daughter strands. Therefore, we focused on H3K4me3 eSPAN to study parental histone H3–H4 segregation.

**Figure 7.**
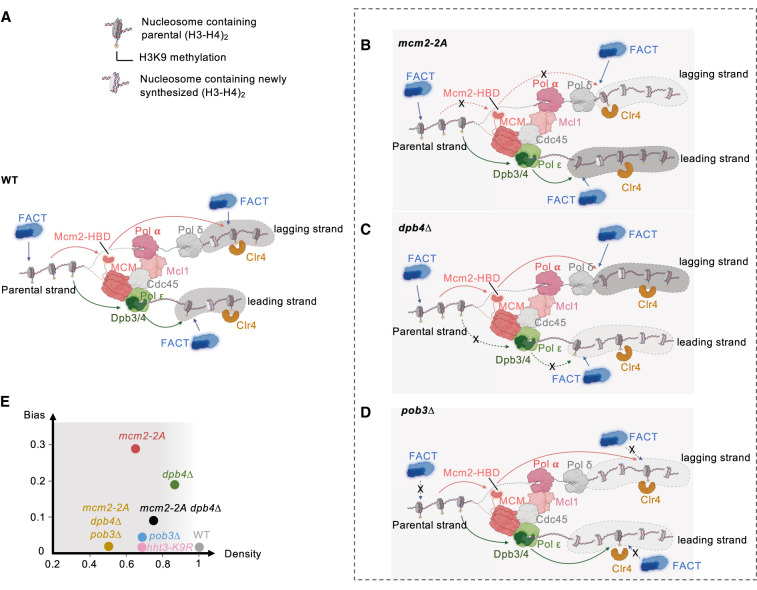
Model of the cooperation of histone chaperones in regulating parental histone segregation to daughter DNA strands and epigenetic inheritance of heterochromatin. (*A*) Schematic diagram illustrating parental histone H3–H4 deposition pathways during DNA replication in wild-type cells. (*B*) Impaired parental histone H3–H4 deposition to the lagging strand in *mcm2-2A* cells leads to a high H3K4me3 eSPAN bias and low H3K4me3 density on the lagging strand. (*C*) Impaired parental histone H3–H4 deposition to the leading strand in *dpb4Δ* cells results in an intermediate H3K4me3 eSPAN bias and a moderate decrease in H3K4me3 density on the leading strand. (*D*) Impaired parental histone H3–H4 deposition to both strands in *pob3Δ* cells causes a low H3K4me3 eSPAN bias and reduced H3K4me3 density on both daughter strands. (*E*) Plotting H3K4me3 eSPAN bias and H3K4me3 density on the lower-density strand. The gradients of gray indicate the severity of the silencing defects.

Our H3K4me3 eSPAN analysis revealed that *mcm2-2A* cells display a leading strand bias, while *dpb4Δ* cells exhibit a lagging strand bias. These results are consistent with previous studies in budding yeast and mammals (Gan et al. 2018; Petryk et al. 2018; Yu et al. 2018; Li et al. 2020; Wen et al. 2023; Wenger et al. 2023), suggesting a highly conserved mechanism of parental histone H3–H4 segregation (Fig. 7A–C). Interestingly, *mcm2-2A dpb4Δ* cells show an intermediate level of both parental histone H3–H4 segregation bias and heterochromatin inheritance. These results are consistent with the idea that a symmetric distribution of parental histone H3–H4 is important for heterochromatin inheritance.

In addition to the symmetric distribution of parental histone H3–H4, our subsequent analyses indicate that heterochromatin inheritance outcomes also depend on parental histone H3–H4 density on daughter strands (Fig. 7D). This is because while a high bias consistently indicates low density on one strand, a low bias fails to differentiate between high density on both strands or low density on both strands. We propose that the relationship between parental histone H3–H4 density and heterochromatin inheritance is akin to the “weakest link theory,” where each daughter strand represents an individual “link” of epigenetic inheritance, and the strand with the lower parental histone levels dictates the inheritance efficiency (Fig. 7E). The profound heterochromatin inheritance defects in *mcm2-2A* cells correspond with the notably low density of parental histone H3–H4 on its lagging strand, whereas the mild defects in *dpb4Δ* cells align with a slight density decrease on its leading strand. Our results are consistent with earlier studies that suggest that low histone turnover rates are critical for epigenetic inheritance (Reddy et al. 2011; Holla et al. 2020; Shan et al. 2020; Cutter DiPiazza et al. 2021), and our model extends this understanding by proposing a discernible threshold of inherited parental histone H3–H4 density that determines the efficiency of epigenetic inheritance.

Heterochromatin formation involves positive feedback loops where, for instance, the H3K9me3-Clr4 “read–write cycles” not only duplicate heterochromatin during DNA replication but also promote heterochromatin spreading (Zhang et al. 2008). These positive feedback cycles have the potential to transform subtle gradient changes into binary switches. When the parental histone H3–H4 density falls below a certain threshold, cells become more susceptible to losing epigenetic information. This prediction is substantiated by the introduction of the H3K9R mutant histone, which effectively reduces the proportion of parental histones containing H3K9me3. Notably, when H3K9R constitutes only 18% of total histone H3, cells can maintain heterochromatin, albeit less effectively than in wild-type cells (Fig. 5F). However, with an increase of H3K9R levels to 36% of total histone H3, a more pronounced loss of heterochromatin occurs, consistent with the existence of a threshold of parental histone H3–H4 density required for epigenetic inheritance (Fig. 5D,F).

It is intriguing to observe that the parental histone H3–H4 density on the lagging strand in *mcm2-2A dpb4Δ* cells is higher than that in *mcm2-2A* cells. This result suggests that the deposition of histones by Mcm2 and Dpb3/4 is competitive. In *mcm2-2A* cells, deposition to the lagging strand fails to compete with Dpb3/4-mediated deposition on the leading strand, resulting in a substantial reduction in lagging strand parental histone H3–H4 density (Fig. 5A). Dpb3/4 captures a portion of released parental histone H3–H4 originally destined for Mcm2 and deposits them to the leading strands. The modest increase in parental histone H3–H4 on the leading strand may be attributed to the fact that Dpb3/4 is either nearing its capacity or facing competition with the deposition of newly synthesized histone H3–H4. However, the simultaneous impairment of Mcm2 and Dpb3/4 recalibrates this balance. First, vacancies emerge for histone H3–H4 deposition in both strands due to the loss of two major parental histone H3–H4 segregation pathways. Second, a larger pool of parental histone H3–H4 becomes available, allowing their incorporation into both strands with the assistance of alternative parental histone segregation pathways. Consequently, parental histone H3–H4 can be recycled on both strands, although with less efficiency than in wild-type cells. This scenario could result in a higher density of parental histone H3–H4 on the lagging strand in *mcm2-2A dpb4Δ* cells compared with *mcm2-2A* cells. However, we could not exclude the possibility that the apparent increase in histone density in double mutant cells is due to normalization artifacts.

Given the established roles of FACT in regulating histone dynamics during DNA replication and heterochromatin inheritance, we investigated its potential contribution to parental histone H3–H4 segregation (Lejeune et al. 2007; Formosa and Winston 2020; Holla et al. 2020; Murawska et al. 2021; Takahata et al. 2021). Indeed, we found that a mutation in FACT (*pob3Δ*) results in only a minor leading strand H3K4me3 eSPAN bias yet elicits a profound impact on H3K4me3 density on both strands. Moreover, *mcm2-2A dpb4Δ pob3Δ* cells show a greater loss of silencing compared with *mcm2-2A dpb4Δ* or *pob3Δ* cells alone, suggesting redundancy in their functions.

It is worth noting that *pob3Δ* is unlikely to entirely abolish FACT function, given the nonessential nature of Pob3 compared with the essential status of Spt16. Therefore, FACT might play a crucial role in all mechanisms of parental histone H3–H4 transfer, with Mcm2 or Dpb3/4 guiding strand-specific parental histone H3–H4 transfer. Moreover, *mcm2-2A* harbors point mutations within the HBD, leaving the possibility of residual histone binding activity, and it is plausible that *dpb4Δ* or *dpb3Δ* indirectly enhances the activity of Mcm2-HBD. Furthermore, the H3K4me3 eSPAN bias levels of *dpb4Δ* are smaller than those of *mcm2-2A*. Despite *dpb3Δ* and *dpb4Δ* being null mutations, there may be other histone chaperone activities that collaborate with Dpb3/4 to facilitate parental histone transfer to the leading strand.

Mcm2, Dpb3/4, and FACT are the major histone H3–H4 chaperones with established roles in regulating parental histone segregation. Here we demonstrate that they have distinct properties: While Mcm2 and Dpb3/4 guide parental histone H3–H4 to the lagging and leading strands, respectively, FACT primarily maintains parental histone H3–H4 to both strands. These cooperative actions collectively ensure the disposition of critical levels of parental histone H3–H4 onto both daughter strands, thus safeguarding the fidelity of epigenetic inheritance.

## Materials and methods

### Fission yeast strains and genetic analyses

Yeast strains containing *mcm2^+^-FLAG* and *mcm2-2A-FLAG* were generated by a PCR-based module method. The FLAG epitope tag was integrated at the endogenous *mcm2*^+^ locus so that the transgene was under the control of its native promoters. Deletion strains *dpb3Δ* and *dpb4Δ* were derived from the Bioneer deletion library and confirmed by PCR analysis, and *pob3Δ* was constructed by a PCR-based module method. All other strains were constructed by genetic crosses. A list of fission yeast strains used in this study is in Supplemental Table S1. For serial dilution analysis, 10-fold dilutions of mid-log stage fission yeast cultures were plated on the indicated media and incubated for 3 d at 30°C.

### Flow cytometry

Fission yeast strains with the *tetO-ura4-gfp*^+^ reporter were grown in liquid culture and maintained at mid-log phase. Cells were harvested at the indicated time points after the addition of tetracycline to a final concentration of 2.5 μg/mL. Cells were resuspended in cold PBS (10 mM Na_2_HPO_4_, 1.8 mM KH_2_PO_4_ at pH 7.4, 137 mM NaCl, 2.7 mM KCl). Cold ethanol was added to a final concentration of 70%, and cells were fixed for 15 min on ice. Cells were then washed twice with PBS and resuspended in a flow cytometry tube (Corning 352008). GFP expression levels were measured by FACSCelesta (Becton Dickinson), and excitation was achieved by using an argon laser emission of 488 nm. Data collection was performed using Cellquest (Becton Dickinson), and a primary gate based on physical parameters (forward and side light scatter) was set to exclude dead cells and debris. Typically, 50,000 cells were analyzed for each strain at each time point. Raw data were processed and analyzed with FlowJo (10.6.2; Becton Dickinson).

### Chromatin immunoprecipitation (ChIP)

Log-phase yeast cultures (OD_595_ ∼0.5) were cross-linked with 1% formaldehyde for 20 min at room temperature with shaking. Glycine (125 mM) was added for an additional 5 min to neutralize formaldehyde. Cells were harvested and washed with cold PBS (10 mM Na_2_HPO_4_, 1.8 mM KH_2_PO_4_ at pH 7.4, 137 mM NaCl, 2.7 mM KCl). Cell pellets then were resuspended in ChIP lysis buffer (50 mM HEPES-KOH at pH 7.5, 140 mM NaCl, 1% Triton X-100, 0.1% deoxycholate, 1 mM PMSF). Cold glass beads were added, and the mixture was shaken vigorously in a MiniBeadBeater (Biospec Products) four times for 30 sec each. The lysates were collected and fragmented with a Bioruptor Pico sonication system (Diagenode) for 12 cycles (30 sec on/30 sec off). After the lysates were clarified by centrifuge at 13,000 rpm for 15 min, released chromatin was immunoprecipitated with the corresponding antibodies—H3K4me3 (EMD Millipore 07-473) and H3K9me3 (Active Motif 39161)—overnight at 4°C. The DNA–antibody mixtures were incubated with Protein G agarose beads (Sigma 11243233001) for 2 h at 4°C. Beads were washed twice with ChIP lysis buffer and once each with ChIP lysis buffer containing 0.5 M NaCl, wash buffer (10 mM Tris at pH 8.0, 250 mM LiCl, 0.5% NP-40, 0.5% deoxycholate, 1 mM EDTA), and TE (50 mM Tris at pH 8.0, 1 mM EDTA). Bead-bound chromatin was eluted with TES (50 mM Tris at pH 8.0, 1 mM EDTA, 1% SDS) at 65°C. The cross-linking was reversed by overnight incubation at 65°C. The DNA–protein mixtures were treated with Proteinase K (Invitrogen 10005393) to release the DNA fragments, and DNA was purified by two phenol:chloroform extractions and precipitated by ethanol.

Quantitative PCR (qPCR) was conducted to calculate ChIP enrichment values with primers specific to the indicated heterochromatin regions. qPCR was performed with Luna Universal qPCR master mix (NEB M3003S) in a StepOne Plus real-time PCR system (Applied Biosystems). A list of DNA oligos used is in Supplemental Table S2.

### RNA analysis

Total cellular RNA was isolated from log-phase cells using the MasterPure yeast RNA purification kit (Epicenter) according to the manufacturer's protocol. Quantification with real-time RT-PCR was performed with Power SYBR Green RNA-to-CT one-step kit (Invitrogen 4389986). RNA serial dilutions were used as templates to generate a standard curve of amplification for each pair of primers, and the relative concentration of the target sequence was calculated accordingly. An *act1* fragment served as a reference to normalize the concentration of samples. The concentration of each target gene in the wild type was arbitrarily set to 1 and served as a reference for other samples.

### eSPAN

Yeast strains containing the *cdc25-22* temperature-sensitive mutant were first grown at the permissive temperature (25°C) until the early–mid log phase (OD 0.2∼0.4). Cultures were then incubated for 4 h at 36°C to arrest cells at the G2 phase of the cell cycle. Cells were then shifted to 25°C to allow them to enter the S phase synchronously. BrdU (Sigma-Aldrich B5002) was added to a final concentration of 650 μM at 25 min after the temperature shift. Cells were cross-linked at 60 min after temperature shift with the addition of 1% formaldehyde and incubated for 20 min at 25°C with shaking. Immunoprecipitation was performed as described above in “Chromatin Immunoprecipitation (ChIP).” Cross-linking was reversed by Chelex-100 (Bio-Rad 142-1253).

BrdU IP was conducted as previously described (Hodson et al. 2003; Yu et al. 2018). Briefly, total and ChIP DNA were incubated for 5 min at 100°C and then immediately cooled for 5 min on ice. DNA was diluted 10 times with BrdU IP buffer (PBS, 0.0625% [v/v] Triton X-100) and incubated with BrdU antibody (BD Bioscience 555627) for 2 h at 4°C. Next, Sepharose Protein G beads (Cytiva 17-0618-01) were added and incubated for an additional 1 h at 4°C. The beads were then washed three times with BrdU IP buffer and once with TE. Finally, DNA was eluted with TES and purified with the Qiagen MinElute PCR purification kit (Qiagen 28004). qPCR was used to test the eSPAN with primers specific to *ARS2004* (an early replication origin) and distal locus *ARS2004* + 25 kb. The ssDNA libraries were prepared with xGen ssDNA and low-input DNA library preparation kit (IDT 10009817).

### Protein extraction and Western blot analysis

Log-phase yeast cultures were collected and washed with cold PBS (10 mM Na_2_HPO_4_, 1.8 mM KH_2_PO_4_ at pH 7.4, 137 mM NaCl, 2.7 mM KCl). Cell pellets were then resuspended in ChIP lysis buffer (50 mM HEPES-KOH at pH 7.5, 140 mM NaCl, 1% Triton X-100, 0.1% deoxycholate, 1 mM PMSF). Cold glass beads were added, and the mixture was shaken vigorously in a MiniBeadBeater (Biospec Products). The lysates were mixed with 2× SDS loading buffer and resolved by SDS-PAGE. The proteins were transferred to a PVDF membrane, and Western blot analysis was performed with FLAG (Sigma F7425) or H3 antibodies.

### Sequencing data analysis

The ChIP, BrdU IP, and eSPAN ssDNA libraries were sequenced by paired-end sequencing under Illumina NextSeq platforms at Columbia University Irving Medical Center, supported by the Herbert Irving Comprehensive Cancer Center. The raw reads were first trimmed by Trim Galore! (https://www.bioinformatics.babraham.ac.uk/projects/trim_galore) to remove adapters and low-quality reads and then mapped to the *S. pombe* genome using Bowtie2 (Langmead and Salzberg 2012). PCR duplicate reads were filtered by Sambamba (Tarasov et al. 2015). The genome-wide read coverage on Watson and Crick strands was calculated at the bin of 1 bp using deepTools bamCoverage (Ramírez et al. 2016).

The bias of read coverage between Watson and Crick strands was calculated at each bin surrounding the origins using the formula

$$\mathrm{bias}=\left(\frac{W-C}{W+C}\right),$$

where *W* and *C* are the read coverages on the Watson and Crick strands, respectively. For the calculation of bias, we used the bin of a 100-bp sliding window in the [−10 kb, 10 kb] region surrounding the origins using the code from https://github.com/clouds-drift/eSPAN-bias (Li et al. 2021). For each strain, both the eSPAN bias and the BrdU bias were calculated. To avoid the background influence from BrdU, the eSPAN bias was further normalized with the corresponding BrdU bias by subtraction.

To quantitively compare the eSPAN signals of parental histones for each DNA strand among samples, the raw eSPAN coverage was first normalized to the total number of reads of each sample. To mitigate potential variation stemming from replication synchronization, BrdU samples from the same genotype were used to compute the coefficients at each origin. Subsequently, the signals surrounding each origin [−2500 bp, 2500 bp] in the corresponding eSPAN sample were fitted by the BrdU coefficients. This approach minimized the impact of variation in BrdU incorporation on eSPAN samples, enabling accurate quantification of density. Statistical significance of single-strand density differences between mutants and the wild type was assessed using a two-sample *t*-test.

### Statistical analysis

The statistical test of sequencing data was performed using R software.

### Data and material availability

Sequencing data have been deposited to NCBI GEO under accession number GSE237770.

## Supplementary Material

Supplement 1

Supplement 2
