# Supplementary material for: Coordination of histone chaperones for parental histone segregation and epigenetic inheritance
Source: Genes Dev. 2024 Feb 1;38(3-4):189–204. doi: 10.1101/gad.351278.123 (PMC10982699; doi:10.1101/gad.351278.123)
Supplement: Supplement 1 [file Supplementary_Figures.pdf]

Figure S1

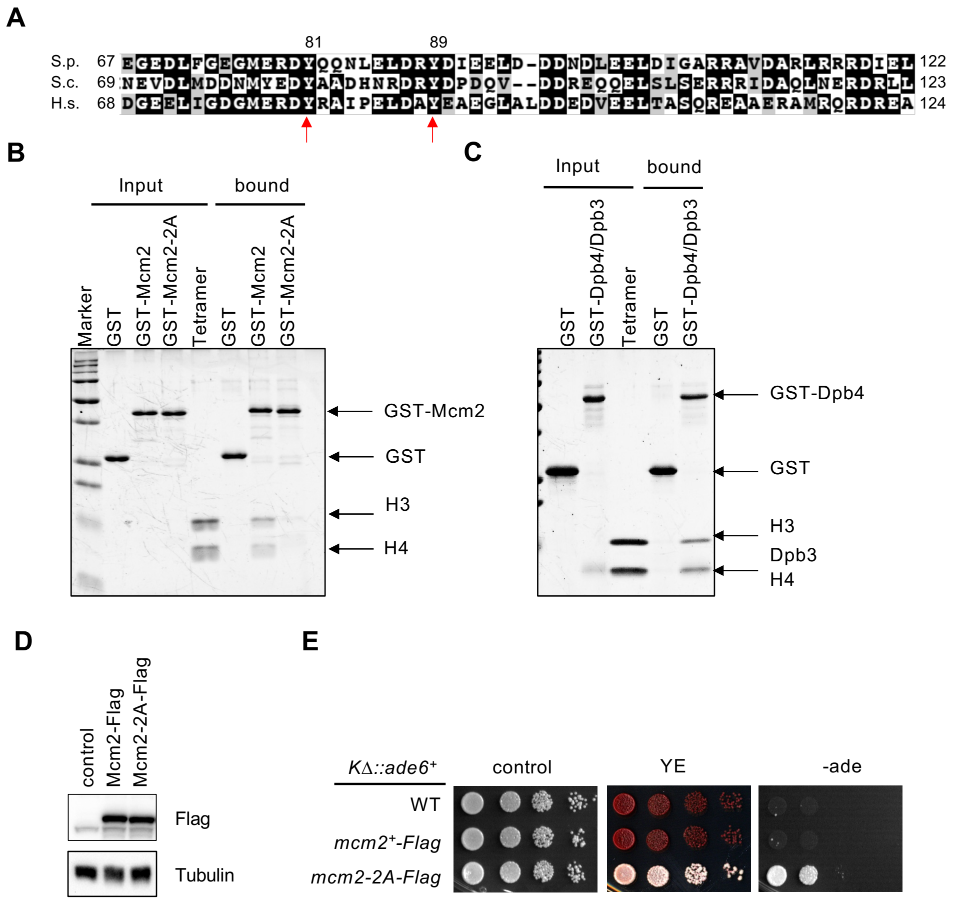

Figure S1. Mcm2 and Dpb3/4 interact with histone H3-H4 tetramers

(A) Sequence alignment of Mcm2-HBD from different species. Red arrows indicate residues mutated in *mcm2-2A*.

(B) Recombinant fission yeast H3-H4 tetramers were incubated with GST, GST-Mcm2-HBD, or GST-Mcm2-HBD-2A immobilized on glutathione beads and washed extensively. The eluted proteins were resolved by SDS-PAGE and stained with Coomassie blue.

(C) Recombinant fission yeast H3-H4 tetramers were incubated with GST or the GST-Dpb4/Dpb3 complex immobilized on glutathione beads and washed extensively. The eluted proteins were resolved by SDS-PAGE and stained with Coomassie blue.

(D) Protein extracts prepared from indicated yeast strains were resolved by SDS-PAGE and analyzed by western blot with Flag (top) and Tubulin (bottom) antibodies.

(E) Serial dilution analysis of indicated strains to measure the expression of *KΔ::ade6*$^+$.

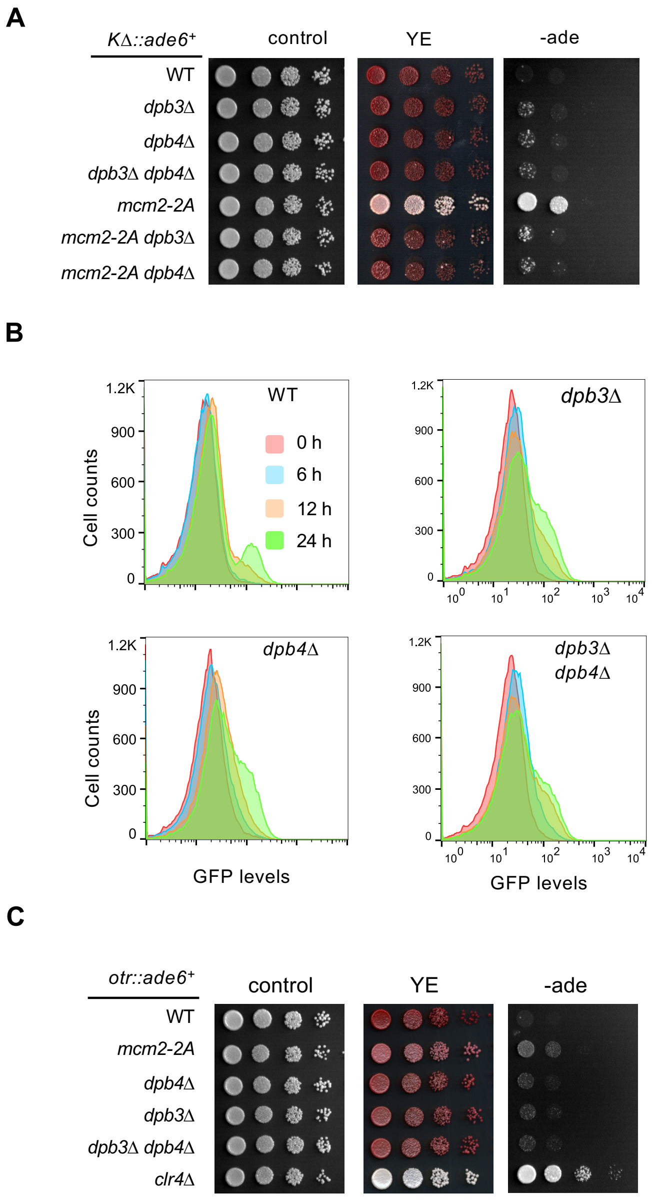

Figure S2. *dpb3Δ* and *dpb4Δ* have similar phenotypes

(A) Serial dilution analysis of indicated strains to measure the expression of *KΔ::ade6*+.

(B) Flow cytometry analysis of GFP expression at different time points after tetracycline addition. All strains are in an *epe1Δ* background.

(C) Serial dilution analysis of indicated strains to measure the expression of *otr::ade6*+.

Figure S3

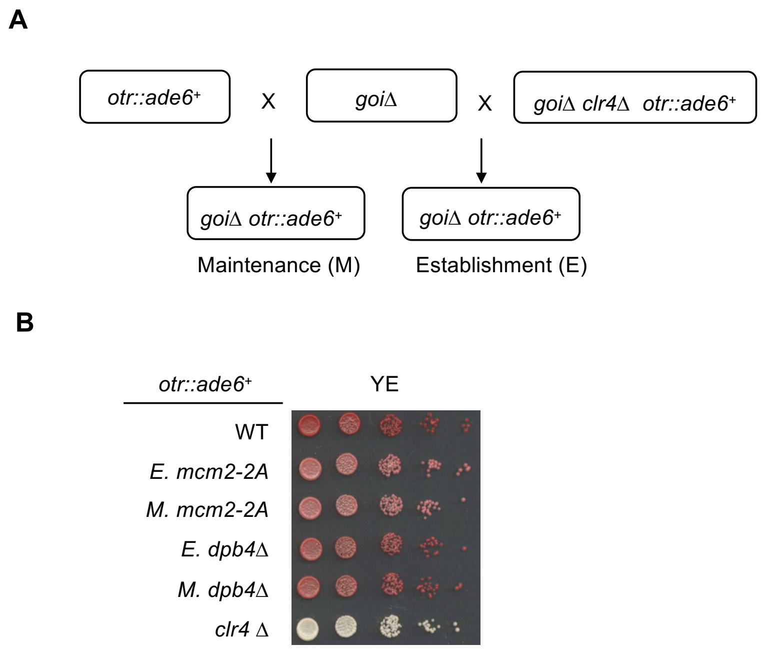

Figure S3. Mcm2 and Dpb4 are not required for heterochromatin establishment
(A) Diagram of genetic crosses to obtain establishment (E) and maintenance strains (M).
(B) Serial dilution analysis of indicated strains to measure the expression of *otr::ade6*$^{+}$.

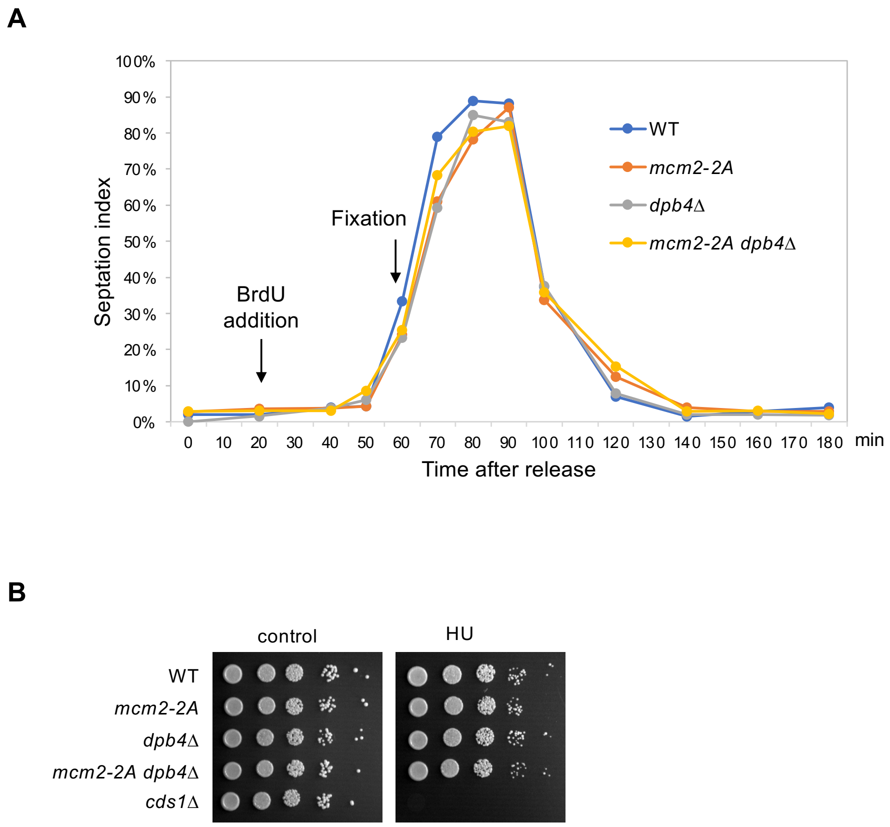

Figure S4. Mcm2 and Dpb3/4 mutants do not affect S phase progression.

(A) The septation index of indicated yeast strains was determined following synchronization with the *cdc25-22* temperature sensitive mutant. Cells were cultured at 36°C to arrest them at G2/M, then shifted to 25°C (time 0) to enter the cell cycle. BrdU was added at 20 minutes and cells were fixed at 60 minutes, corresponding to early S phase.

(B) Serial dilution analysis of indicated strains to measure their sensitivity to 5 mM of HU.

A

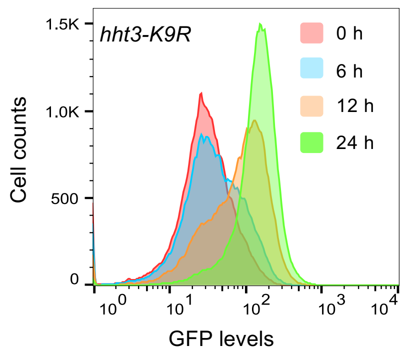

B

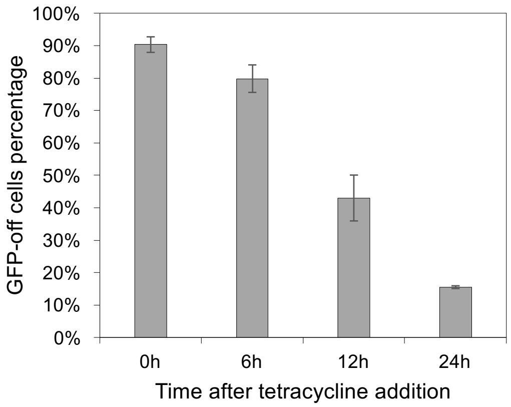

Figure S5. Hht3-K9R affects heterochromatin inheritance

(A) Flow cytometry analysis of GFP expression at different time points following tetracycline addition. All strains are in an *epe1Δ* background.

(B) Quantification of the percentage of cells maintaining low GFP expression at different time points after tetracycline addition. Data are presented as mean ± SD of two biological replicates. All strains are in an *epe1Δ* background.

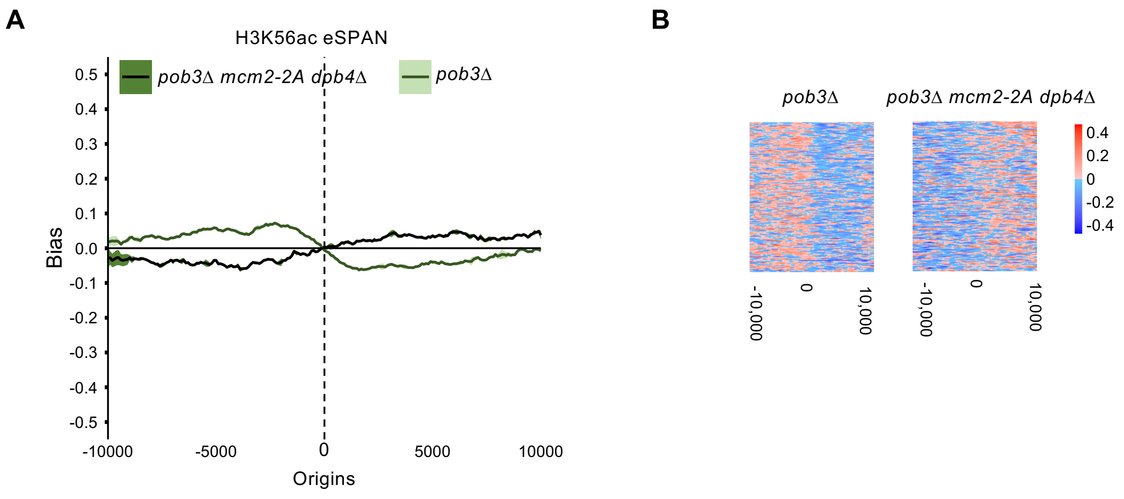

Figure S6. H3K56ac eSPAN of *pob3Δ* mirrors H3K4me3 eSPAN

(A) eSPAN analysis of H3K56ac bias levels at replication origins. The shading of the bias line plot is the 95% confidence interval of mean value of at least two biological replicates, which is mean ± 2 folds of the standard error.

(B) Heatmaps of H3K4me3 bias at each of the 162 replication origins analyzed.
